# Inflammatory cytokines and sarcopenia in Iranian adults-results from SARIR study

**DOI:** 10.1038/s41598-022-09139-3

**Published:** 2022-03-31

**Authors:** Farzaneh Asoudeh, Fatemeh Dashti, Shima Raeesi, Ramin Heshmat, Mohammad Bidkhori, Zahra Jalilian, Rezvan Hashemi

**Affiliations:** 1 Department of Clinical Nutrition, School of Nutritional Sciences and Dietetics, Tehran University of Medical Sciences, Tehran, Iran; 2 Department of Geriatric Medicine, Ziaeian Hospital, Tehran University of Medical Sciences (TUMS), Abuzar St., P.O. Box 14155-6117, Tehran, Iran; 3 Endocrinology and Metabolism Research Center and Chronic Disease Research Center, Tehran University of Medical Science, Tehran, Iran; 4 Department of Epidemiology and Biostatistics, School of Public Health, Tehran University of Medical Sciences, Tehran, Iran; 5 Rajaie Cardiovascular Medical and Research Center, Iran University of Medical Sciences, Tehran, Iran

**Keywords:** Health care, Medical research

## Abstract

Some studies suggested the effects of inflammatory cytokines in reducing muscle mass and muscle strength and, performance. This study aimed to compare pro-inflammatory cytokines in sarcopenic and non-sarcopenic subjects. 120 men and women were selected out from the cross-sectional study ‘sarcopenia and its determinants among Iranian elders’ (SARIR). Sarcopenia was defined based on the first ‘European Working Group on sarcopenia in older people’ (EWGSOP_1_) guidelines. A fasting blood sample was taken from each participant to measure serum high-sensitivity C-reactive protein (hs-CRP), Interleukin 6 (IL-6), and tumor necrosis factor α (TNFα). A total of 120 participants were included in this study. Mean age was 66.7 ± 7.7 years and mean body mass index (BMI) was 27.3 ± 4.2 kg/m^2^. Forty participants had the criteria of EWGSOP_1_ sarcopenia. A statistically significant difference was seen between normal and abnormal groups of muscle strength in hs-CRP (P-value = 0.04). Furthermore, we did not observe any remarkable association between inflammatory biomarkers including IL-6 (OR 1.15; 95% CI 0.31–4.28), TNF-α (OR 0.68; 95% CI 0.17–2.77), and hs-CRP (OR 2.39; 95% CI 0.87–6.55) and the presence of sarcopenia even after controlling for plausible confounders. We found that inflammatory biomarkers level was not associated with odds of sarcopenia. The lack of correlation between inflammatory cytokines and sarcopenia could be due to the participants’ age and genetics. Future studies are required to confirm these findings.

## Introduction

The proportion of elderly is rapidly increasing in World’s population^1^. Old age is associated with reduced physical function with negative impacts on quality of life and activities of daily living^2^. Sarcopenia is characterized by skeletal muscle loss, low muscle strength and/or physical performance and is one of the critical issues in geriatric medicine^3^. Based on the definition provided by the European Working Group on sarcopenia (EWGSOP_1_), sarcopenia is the presence of both low muscle mass and low muscle function (including strength or performance)^4^. Besides being common among elderly people, sarcopenia can also occur in early life as a consequence of other inflammatory conditions and systemic diseases which is called secondary sarcopenia, due to the involvement of causal factors other than (or in addition to) aging^5^. Other factors that potentially may play a role in the development of sarcopenia are poor nutrition and physical inactivity^5^. Sarcopenia can result in disabilities and an increased risk of frailty, falls, fractures, and other consequences that burden society and health care services^6–8^.

Some physiological changes related to aging, including oxidative stress, abnormal anabolic hormone production, neuromuscular atrophy, and inflammatory factors, are associated with decreased muscle mass and strength^9^. High levels of inflammatory factors have been associated with an increased odds of disability^10, 11^ and mortality^12^ in older adults. On the other hand, increased plasma levels of pro-inflammatory cytokines and acute-phase proteins are often observed in older sarcopenic populations^13^. There are various hypotheses proposed to examine the effect of inflammation on physical performance and muscle strength^2^. Previous studies have suggested that inflammatory mediators could induce muscle wasting by affecting muscle protein metabolism^14, 15^. In line with this, other studies have shown that increasing levels of inflammatory markers such as IL-6, TNF-a, and CRP in older adults are associated with increased muscle loss and progression of sarcopenia^16–18^. Schaap et al. in the ‘Longitudinal Aging Study Amsterdam’ indicated that higher levels of serum IL-6 and CRP were associated with an increased risk of muscle strength loss during 3 years of follow-up^17^. Moreover, Taaffe et al. in a cross-sectional and prospective study, indicated a reverse relationship between CRP level and handgrip strength, though high baseline concentrations did not predict a 7-year later decrease in performance and grip strength^19^. Tuttle et al. showed in a recent systematic review and meta-analysis that higher levels of inflammatory markers including IL-6, TNF-a, and CRP were associated with decreased skeletal muscle mass and muscle strength^20^. Most previous studies on the relation of inflammatory markers and sarcopenia have been conducted in Europe and the USA. There have been only few studies in Asian populations^21–23^. On the other hand, prevalence of sarcopenia among Asia population^24^ is higher than population in western coutries^25^ and number of elderly population in Iran is growing. In this cross-sectional study, we aim to compare inflammatory factors levels, including IL6, CRP, and TNFα, between sarcopenia and non-sarcopenia in Iranian adults. In addition we evaluated the relationship between inflammatory factors and sarcopenia components including muscle performance, strength, and muscle mass in the Iranian elder population.

## Materials and methods

Phase one of this population-based cross-sectional study was carried out between May and October 2011 in Tehran, Iran. Three hundred men and women were invited randomly from the 6th district of Tehran. The details of the sampling method and data collection procedure of the SARIR study were reported previously^26^. After assessment of muscle mass, strength and performance, 54 participants were identified as sarcopenic.

In phase two, 40 sarcopenic and 80 non-sarcopenic individuals were randomly selected out—due to the limited budget—based on participant’s codes. Then inflammatory markers were measured for them.

This study was performed according to the Declaration of Helsinki. The study protocol was accepted by the TUMS (Tehran University of Medical Sciences) ethics committee. All subjects confirmed the written informed consent before being included in the study.

### Assessment of muscle mass

DXA scanner (Discovery W S/N 84430), which can measure fat, muscle, and bone mass of limbs, was used to determine body composition for each person. According to DXA results, we calculated the appendicular skeletal muscle for each participant as the sum of upper and lower limb muscle mass (in kg)^27^.

We calculated the skeletal muscle mass index by dividing the sum of muscles’ limbs to height square^28^. Appendicular skeletal muscle index less than 7.26 kg/m^2^ for men and less than 5.45 kg/m^2^ for women were considered abnormal^4^.

### Assessment of muscle strength

Handgrip test was done using a pneumatic instrument—a squeeze bulb dynamometer (manufactured by Jamar, Inc. USA: c7489-02 Rolyan) calibrated in pounds per square inch (psi)—to measure the muscle strength. Participants were requested to sit in a straight-backed chair, with their shoulders adducted in a neutral position, arms unsupported, and elbows flexed at 90°. The measurement of maximum voluntary contraction (handgrip strength) was done three times with 30 s rest in between measurements for each hand. The average measurements of the subjects’ hands were defined as muscle strength. To identify low muscle strength for each participant, age and sex-specific thresholds suggested by Merkies et al. were used^29^.

### Assessment of muscle performance

We asked each individual to walk at his/her usual pace to measure the muscle performance using a 4-m walk gait speed test. Subjects with gait speeds lower than 0.8 m/s were identified as having abnormal muscle performance.

### Sarcopenia determination

The current study used European Working Group on Sarcopenia in Older People (EWGSOP_1_) guidelines for sarcopenia definition^4^.

A sarcopenic person is an individual with abnormal hand grip strength or abnormal gait speed test in addition to abnormal muscle mass.

### Assessment of biomarkers

Blood samples were taken after 12-h fasting. Serum samples were kept at − 80 °C until performing the ELISA test in the Endocrinology and Metabolism Research Center of Tehran University in specialized laboratories. The ELISA method was used to measure serum IL-6 and TNF-α levels using commercial kits (I.D. labs Canadian company), and the hs-CRP test was performed using Pars Azmoon kit. All the assessments were done in duplicate for all inflammatory cytokine measures. More details were reported previously^26^.

### Assessment of other variables

A short form of the International Physical Activity Questionnaire (IPAQ) was used to assess the physical activity level based on metabolic equivalent hours per week (MET-h/week). A digital scale was used for the body weight of the participants only wearing minimal clothing. Height was assessed using a wall tape measure with the participants in a standing position without shoes. Body mass index (BMI) was computed by dividing weight (kg) by height squared (m_2_).

### Statistical analysis

Normally distributed variables were described using mean and standard deviation. The general characteristics of the study individuals were compared using one-way analysis of variance and chi-squared tests. The distribution of inflammatory markers was examined using the Kolmogorov–Smirnov test. Since plasma levels of inflammatory markers were not normally distributed, the analyses were performed using log-transformed values. T-test and Mann Whitney test were used to compare the hs-CRP, IL6, and TNFα levels between normal and abnormal muscle mass, handgrip strength, and gait speed. Multivariate logistic regression was applied to find the relationship between inflammatory cytokines and the presence of sarcopenia. In the first statistical model, we adjusted for age (years) and sex (male/female). Further controlling was performed for physical activity smoking (yes/no), (MET-h/week), alcohol consumption (yes/no), medication use (yes/no), and history of the disease (yes/no) in the second model. hs-CRP, IL-6, and TNF-α were categorized based on detection limits (5 mg/l for hs-CRP, 10 pg/ml for IL-6, and 8 pg/ml for TNF-α), which levels above the detection limits identified as an abnormal. Our statistical analyses were performed using SPSS software Version 16 (SPSS Inc., Chicago, IL, USA). P values less than 0.05 were considered statistically significant.

### Research ethics and patient consent

The study protocol was approved by the Tehran University of Medical Sciences ethics committee. We explained the study’s aims to the participants at first and then requested all participants to complete a written informed consent before data collection.

## Results

A total of 120 participants were included in this study. General characteristics of participants are presented in Table 1. About 50% of the participants in both the sarcopenia group and the non-sarcopenia group were female. Only 28% of the sarcopenia group had a medical history (including diabetes, myocardial infarction, cerebrovascular accident, asthma and arthritis) versus 40% in the non-sarcopenia group. Regarding taking medication, 5% of sarcopenia and 2.5% of the non-sarcopenia group were using sexual hormones, and 2.5% of both groups were taking corticosteroids. In the sarcopenia group, handgrip strength was significantly lower than the control group.

**Table 1:** Characteristic of the study participants.

	Sarcopenia (N = 40)	Non-sarcopenia (N = 80)	P-value
Age (year)	67.1 ± 7.9	66.6 ± 8/1	0.67**
Weight (kg)	62.4 ± 11.2	73.8 ± 12.1	0.76
Height (cm)	160.4 ± 9.5	161 ± 9	0.50
Body mass index (kg/m^2^)	26.8 ± 17.7	28.2 ± 4.4	0.18
Handgrip strength (Psi)	9.6 ± 2.9	11.4 ± 3.8	0.03
4-m gait speed test (m/s)	0.78 ± 0.2	0.84 ± 0.2	0.08
Appendicular skeletal muscle (kg)	15.6 ± 4.6	17.8 ± 3.8	0.54
Muscle Index (kg/m^2^)*	8.2 ± 14.9	6.7 ± 0.9	0.38
Physical activity (met. h/week)	1260.7 ± 1450.5	1140.6 ± 1034.1	0.60
Marital status (%married)	85	78.8	0.08
Gender (%female)	50	48.8	0.89
Education status (%)			
**History of alcohol use in past 6 months**			0.55
Never (%)	85	88.8	
Sometimes (%)	15	11.3	
**History of cigarette smoking use in past 6 months**			0.44
Never (%)	85	83.3	
Always (%)	15	12.5	
Medical history**			0.17
No (%)	72.5	60	
Yes (%)	27.5	40	
**Drug history**			
Sexual hormone use (%)	5	2.5	0.47
Statin use (%)	42.5	32.5	0.28
Corticosteroid use (%)	2.5	2.5	1.00

Values are mean ± S.D. unless otherwise stated.

*Muscle index = appendicular skeletal muscle/height^2^.

**Pvalue < 0.05 significant.

**Medical history including diabetes, myocardial infarction, cerebrovascular accident, asthma and arthritis.

Comparison of inflammatory cytokines between normal and abnormal components of sarcopenia, including handgrip strength, muscle mass, and gait speed test, is shown in Table 2. These results showed a statistically significant difference in serum hs-CRP between normal and abnormal handgrip strength groups (P-value = 0.04). There was no statistically significant difference in other groups. Logistic regression analysis of the relationship between inflammatory biomarkers and presence of sarcopenia is represented in Table 3. In the crude model, there was no association between hs-CRP levels and risk of sarcopenia (OR 1.02; 95% CI 0.32, 3.24). In addition, after adjustment for plausible confounders, this association remained non-significant (OR 1.15; 95% CI 0.31, 4.28). Moreover, we did not observe any association between IL-6 and TNF-α and presence of sarcopenia neither before (OR 0.90; 95% CI 0.25, 3.13, OR 1.60; 95% CI 0.73, 3.48) nor after controlling for potential confounders (OR 0.68; 95% CI 0.17, 2.77, OR 2.39; 95% CI 0.87, 6.55).

**Table 2:** Comparison of inflammatory cytokine between normal and abnormal muscle mass, handgrip strength, and gait speed.

Inflammatory factor	Muscle mass			Handgrip strength			Gait speed test		
	Normal	Abnormal	P*	Normal	Abnormal	P*	normal	Abnormal	P*
	N = 57	N = 63		N = 72	N = 48		N = 63	N = 57	
hs-CRP (mg/dl)	2.55 ± 2.72	4.01 ± 11.06	0.1	2.6 ± 0.4	3.7 ± 1.2	0.04	2.6 ± 0.4	11.2 ± 1.5	0.6
TNFα (Pg/dl)	33.2 ± 9.2	34.8 ± 9.6	0.8	21.4 ± 4.5	42.1 ± 10.4	0.5	36.5 ± 10.0	31.0 ± 8.5	0.6
IL6 (Pg/dl)	10.7 ± 0.3	10.6 ± 0.3	0.3	10.6 ± 0.4	10.7 ± 0.2	0.8	10.6 ± 0.3	10.8 ± 0.3	0.2

All values are mean ± SD.

*P-value < 0.05 significant.

**Table 3:** Multivariable-adjusted odds ratios (95% C.I.s) for sarcopenia and inflammatory biomarkers.

	Crude		Model 1		Model 2	
	OR** (95% CI)	P_value_	OR (95% CI)	P_value_	OR (95% CI)	P_value_
CRP	1.02 (0.32, 3.24)	0.96	1.00 (0.31, 3.17)	0.99	1.15 (0.31, 4.28)	0.82
IL-6	0.90 (0.25, 3.13)	0.87	0.91 (0.26, 3.17)	0.88	0.68 (0.17, 2.77)	0.59
TNF-α	1.6 (0.73, 3.48)	0.23	1.69 (0.76, 3.75)	0.19	2.39 (0.87, 6.55)	0.08

Sarcopenia was defined based on the European Working Group on sarcopenia in older people 2 (EWGSOP_1_) definition.

Model 1: Adjusted for age (continuous), sex (male/female).

Model 2: Further adjusted for physical activity, smoking, alcohol consumption, drug history, and history of the disease (including diabetes, myocardial infarction, cerebrovascular accident, asthma and arthritis).

**The analysis of binary logistic regression was used to determine the OR and 95% confidence interval.

## Discussion

In the current study, we investigated the association between the presence of sarcopenia and inflammatory markers including TNF-a, IL-6, and hs-CRP. No association was found. In addition to this, we investigated the levels of TNF-a and IL-6 between normal and abnormal groups of muscle mass and muscle performance and found no significant interaction. However, a significant difference in hs-CRP level between normal and abnormal muscle strength groups was observed. Several previous studies showed that inflammation plays a role in the developing of sarcopenia^16, 17, 20^. A longitudinal aging study in Amsterdam revealed a positive association between higher levels of IL-6 and CRP and elevated risk of muscle strength loss but not muscle mass^17^. Moreover, in another prospective cohort study, the researchers found that increased levels of inflammatory markers were significantly correlated with a more 5-year decrease in the thigh muscle area^16^. Higher TNF-α levels and its soluble receptors investigated the most consistent relationship with lower grip strength and muscle mass^16^. In support of these findings, a systematic review and meta-analysis that evaluated the association of markers of inflammation with muscle strength and muscle mass found a significant relationship between increased levels of circulating TNF-α, CRP, and IL-6 with a decline in muscle mass, handgrip and knee extension strength^20^. On the other hand, consistent with our findings, a systematic review and meta-analysis of cross-sectional studies investigated that subjects with sarcopenia compared with controls have higher levels of serum hs-CRP. However, levels of IL-6 and TNF-α were not significantly different^30^. This could be due to the small sample size of the present study. In addition, differences in methodological approach such as control of confounders in the analyses might also contribute.

Overall, TNF-α, CRP, and IL-6 are inflammatory markers that are considered suitable indicators of inflammatory status for investigating the association with sarcopenia and its components^30^. CRP is an indicator of acute and chronic phase inflammation and increases chronic diseases such as type 2 diabetes, cardiovascular disease, and sarcopenia. Muscle mass, muscle strength, and physical performance decline in chronically high level of inflammatory markers such as CRP^30–32^. Increased levels of CRP influences muscle cell size by suppression of the muscle protein synthesis pathway^33^. Furthermore, high levels of CRP are associated with obesity and insulin resistance^34^. It seems that there is a direct relation between the mechanical and metabolic performance of aged muscles^35^. Increasing CRP levels can increase insulin resistance, which disturbs muscles' metabolic function, and as a result, mechanical function is also impaired^35^. Although the reason is unknown, the mechanism of cellular and molecular changes in sarcopenia and insulin resistance are the same^36^. In both cases, there is an accumulation of fat in muscle fibers which can affect the insulin pathway^36^. Furthermore, disruption of a critical protein in muscles, such as myosin heavy chain, can be seen in insulin resistance and aging muscles^37, 38^. However, the exact effect of CRP on muscle atrophy is still unknown^20^.

IL-6 is an Inflammatory cytokine secreted by immune cells in tissue damage or infection conditions^20^. Also, IL-6 is known as a myokine, which is produced by skeletal muscle and regulates muscle contraction and metabolism^39, 40^. Some studies showed that temporal and low levels of IL-6 could be beneficial^41^; however, it is well understood that chronic exposure to IL-6 may result in muscle atrophy and facilitate muscle catabolism^42^. TNF-α is a pro-inflammatory cytokine and has been associated with muscle pathology^43^. Muscle catabolism in various inflammatory diseases, including congestive heart failure^44^, cancer^45^, and chronic obstructive pulmonary disease (COPD)^46^, has been attributed to TNF-α. Studies investigate that TNF-α interferes with the muscle differentiation process and can elevate catabolism in mature cells^47^. Also, TNF-α, through another pathway mediated by reactive oxygen species (ROS) and nuclear factor-kappa B, promotes muscle wasting^47^. Verghese et al. showed in a cross-sectional study that higher levels of IL-6 but not TNF-α were associated with gait speed decline in elderly^48^. Renner et al. showed in another cross-sectional study a negative association between inflammatory markers including IL-6 as well as CRP and functional decline^49^. Along with the catabolic effect of inflammatory markers on muscle mass, as discussed earlier^17, 18^, the negative effect of inflammatory cytokines on the aging brain might lead to motor function disorder and is another possible mechanism in this regard^48, 50^.

Recently, researchers have presented interesting data concerning the hypothesis of a “gut-muscle axis” and sarcopenia. They suggest that disruption of intestinal epithelial tight junctions and gut dysbiosis which especially occur in IBD (inflammatory bowel disease) patients through inflammatory markers could lead to muscle wasting and sarcopenia^51^. Higher levels of inflammatory markers make skeletal muscle fibers initiate an overproduction of reactive oxygen species (ROS)^52^ and finally cause oxidative stress and consequently sarcopenia^53^. This supports the crucial association of inflammatory markers with muscle mass/strength and sarcopenia.

In the present study, we found no significant association between inflammatory markers and sarcopenia and its components, including muscle mass and muscle performance. We have only seen a significant association between normal and abnormal muscle strength groups evaluated based on handgrip strength. The difference between our findings and previous studies could be related to the limited number of participants in this study, and more longitudinal studies are needed to clarify our findings. Also, this inconsistency between our results and previous studies may be due to racial and age differences in the current study.

It may be because of the way that cytokines were measured. The measurement of plasma inflammatory cytokines may not suffice to determine the differences, and cellular cytokines must be measured. Inflammation statuses such as disability, neurodegenerative processes, and aging-related hormonal changes can be other causes of sarcopenia^54^.

To the best of our knowledge, the present study is the first population-based study that evaluated the association of sarcopenia and its components with inflammatory markers in an Iranian population. Our study had some limitations that need to be considered: our study had a small sample size. The causal association between inflammatory markers and sarcopenia cannot be evaluated due to the study’s cross-sectional design. The usage of hydraulic dynamometer is recommended by EWGSOP_1_. However due the limits we used pneumatic dynamometer instead. Which have different measurement units. With regards to using IPAQ, although the validity of this assessment tool is generally considered adequate among elderly, some difficulties including non-repeatability and classification disagreement exist^55^. Finally, we conducted the present study on older adults, so generalization of these results to other age groups should be cautious.

## Conclusion

There was no significant association between TNF-α, IL-6, and hs-CRP with the presence of sarcopenia, nor with muscle mass, and performance. However, we found a significant difference between serum level of hs-CRP and muscle strength. More prospective studies are needed to provide further insights into the association between these inflammatory markers and sarcopenia.
